# Precise endoballoon positioning for High‐Risk sternal re‐entry in an ascending aortic pseudoaneurysm with patent coronary grafts

**DOI:** 10.1111/jocs.17016

**Published:** 2022-10-17

**Authors:** Katie E. O'Sullivan, Anand R. Mehta, Patrick R. Vargo

**Affiliations:** ^1^ Departments of Thoracic and Cardiovascular Surgery The Cleveland Clinic Cleveland Ohio USA; ^2^ Department of Cardiothoracic Anesthesiology The Cleveland Clinic Cleveland Ohio USA

**Keywords:** aorta and great vessels

## Abstract

Redo cardiac surgery can present a unique set of challenges even to the experienced surgeon. Although outcomes have steadily improved in the modern era; if an intraoperative adverse event occurs, there is a 5% incidence of mortality and 19% incidence of myocardial infarction, stroke or death. Overall, the modern incidence of mortality at reoperation varies but be segregated into low and higher risk cohorts depending on the planning computed tomography imaging and risk to substernal structures on re‐entry. Patients with ascending aortic or root pseudoaneurysms represent a particularly difficult subset of high‐risk patients requiring reoperative cardiac surgery due to the danger of exsanguination and air embolization. The gold standard for management of such cases remains the use of deep hypothermic circulatory arrest (DHCA) to achieve safe re‐entry in such cases however this can result in unpredictable DHCA duration depending on the degree of pericardial adhesions. We report a case of aortic pseudoaneurysm in a patient with patent coronary grafts managed using an endoballoon precisely positioned relative to the proximal anastomoses resulting in a safe surgical re‐entry and shorter DHCA time.

## 1 CASE DESCRIPTION

**Figure 1:**
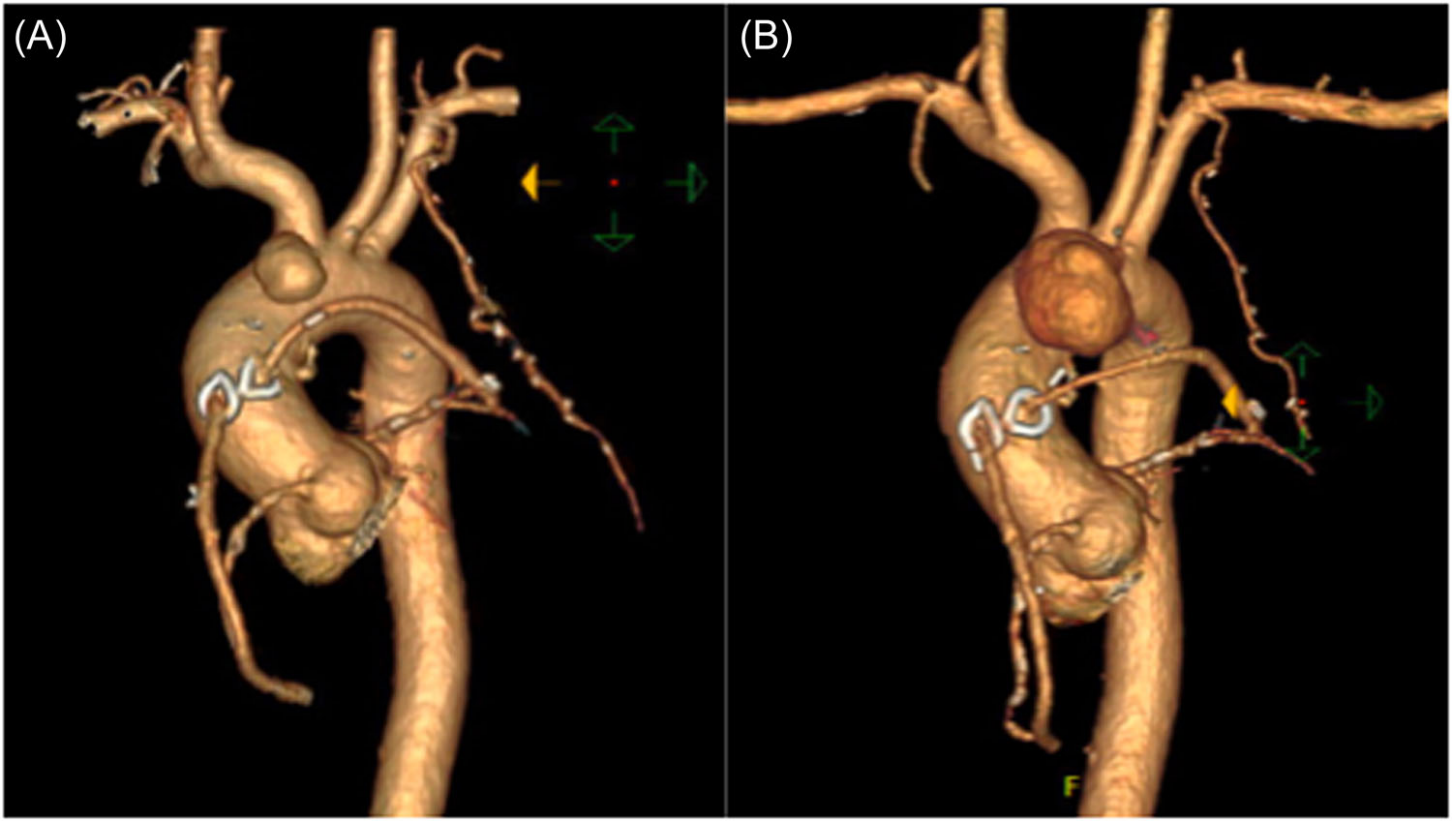
(A) 3D CT reconstruction demonstrating the aortic pseudoaneurysm when initially diagnosed in communication with the former aortic cannulation site. (B) A further 3D CT reconstruction demonstrates enlargement of the pseudoaneurysm observed 12 days later with interval reimaging. CT, computed tomography; 3D, three dimensional.

A 52‐year‐old gentleman, with several cardiovascular risk factors was referred for surgical management; other history was notable for end stage renal disease with a left upper limb fistula, severe peripheral vascular disease, and Protein C deficiency resulting in recurrent deep vein thrombosis requiring a greenfield filter. He had undergone coronary artery bypass grafting at another center 5 months prior with left internal mammary artery to left anterior descending artery (LIMA‐LAD), and saphenous vein grafts to posterior descending artery and ramus intermedius complicated by left arm ischemia requiring angioplasty. He had represented at an interval with progressive dizziness, fatigue, and lightheadedness. Computed tomography (CT) imaging revealed an aortic pseudoaneurysm at the original aortic cannulation site with an ostium of 9 × 7 mm that was compressing 50% of the main pulmonary artery (Figure 1A).^1^, ^2^, ^3^, ^4^, ^5^

**Figure 2:**
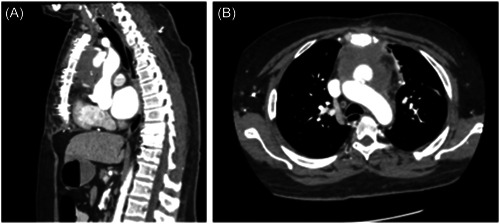
(A) Sagittal section demonstrating the enlarging aortic pseudoaneurysm. Of note is the relationship of the pseudoaneurysm neck in relation to the right main pulmonary artery on this image. This relationship was used as an anatomical landmark which was constant between CT and TEE imaging. This landmark was used to position the endoballoon intraoperatively as we knew the patent coronary grafts were more proximal (B) Axial CT images showing the proximity of the pseudoaneurysm to the posterior sternal table highlighting the risk of rupture upon re‐entry. CT, computed tomography; TEE, transthoracic echocardiogram.

In addition to his remaining workup, an interval CT 12 days later demonstrated an increase in the size of the pseudoaneurysm and surrounding complex fluid collection in the anterior mediastinum. Infectious etiology could not be out‐ruled (Figure 1B, Figure 2).

**Figure 3:**
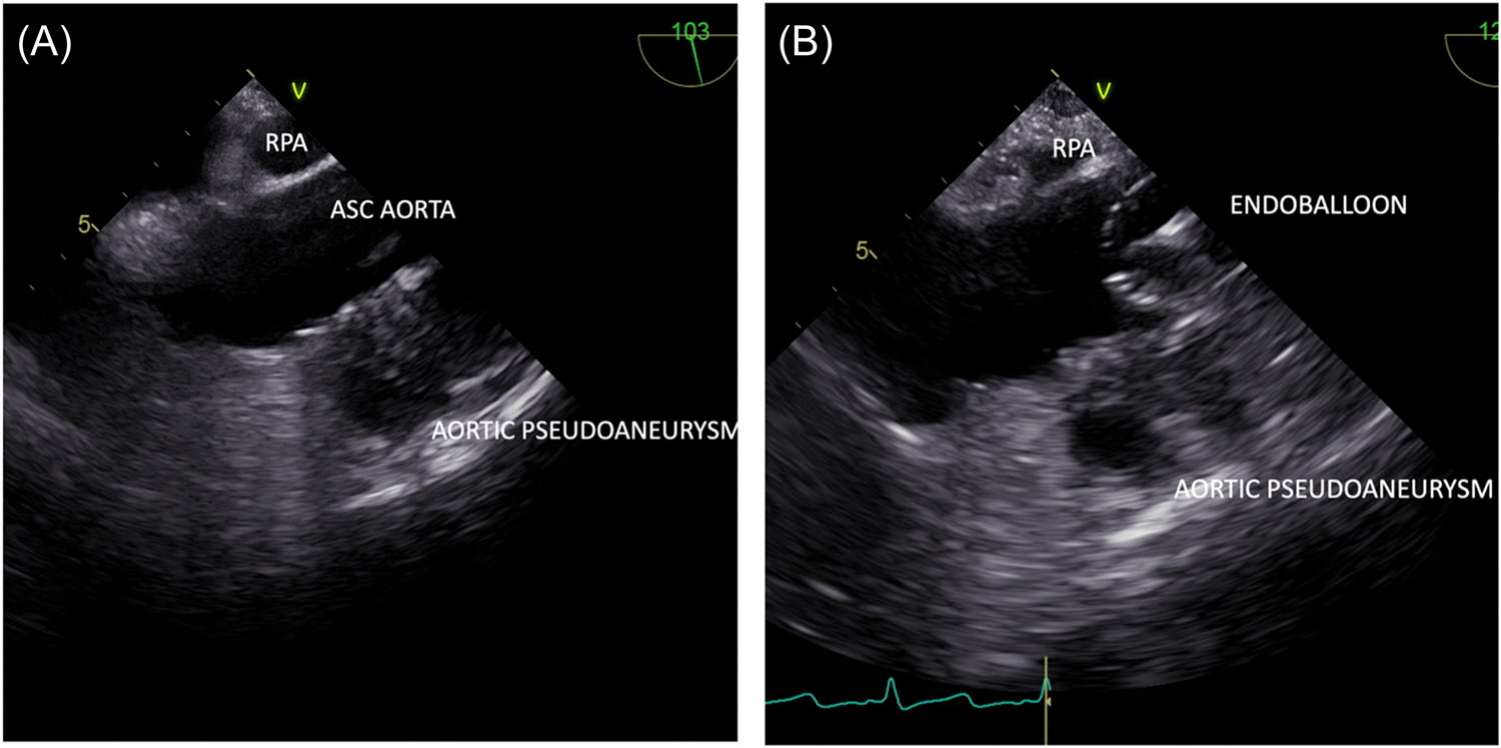
(A) TEE image demonstrating preoperative aortic pseudoaneurysm along the anterior aspect of the ascending aorta, the right main pulmonary artery was utilized as a landmark for endoballoon positioning safely above the patent proximal coronary anastomoses (B) Intraoperative image demonstrating the inflated endoballoon in situ above the patent grafts while cardioplegia was being delivered to the aortic root. TEE, transthoracic echocardiogram

Surgery was carried out using right axillary cannulation with a Y to the right common femoral artery. A venous access cannula was placed in the right femoral vein. An endoballoon was advanced from the left femoral artery to the distal ascending aorta just distal to the aortocoronary grafts using a combination of fluoroscopy and transthoracic echocardiogram (TEE) (Figure 3).

**Figure 4:**
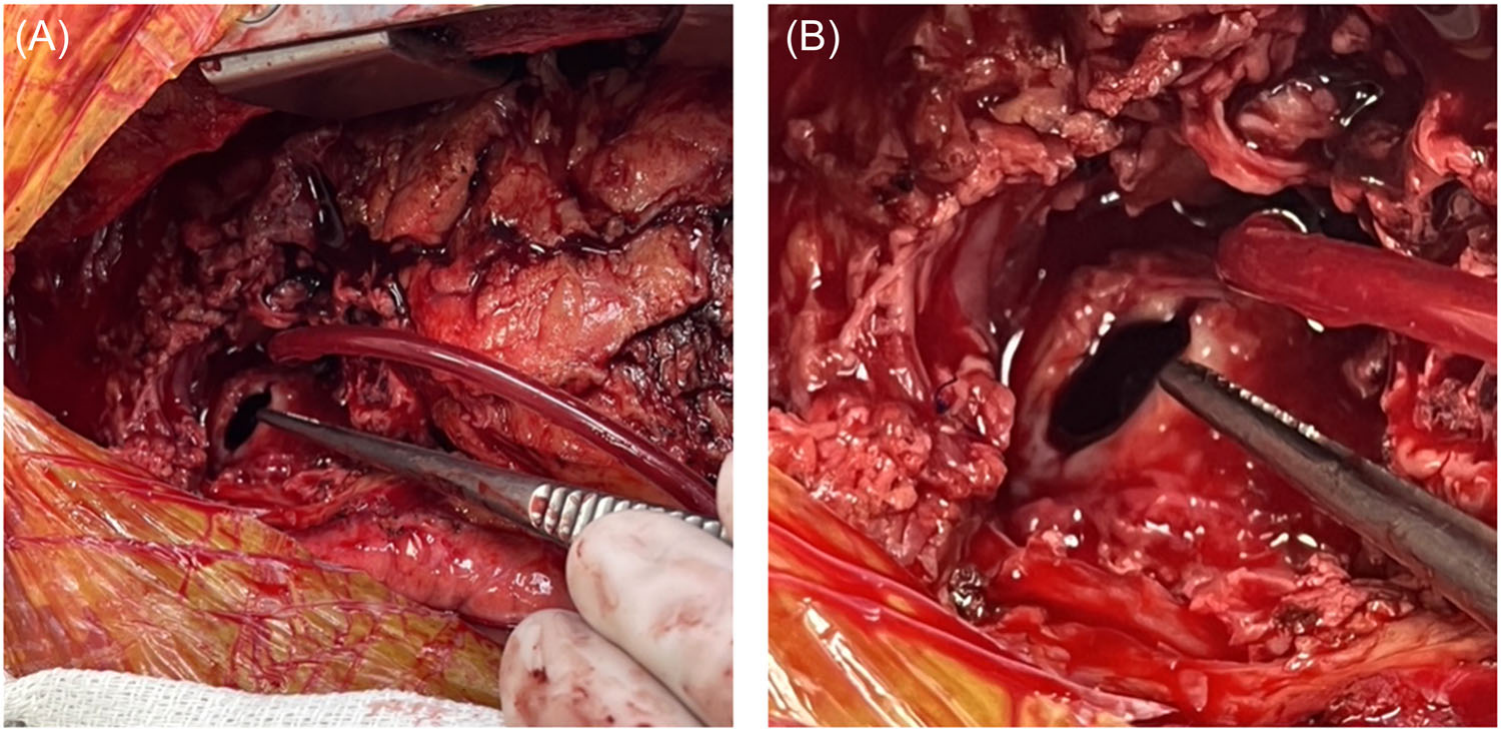
(A, B) these intraoperative images demonstrate the 1 cm defect in the ascending aorta, shown at the tip of the forceps, which was subsequently repaired

A percutaneous retrograde cardioplegia catheter was advanced under TEE guidance into the coronary sinus. Cardiopulmonary bypass was commenced and upon resternotomy bleeding was encountered from the pseudoaneurysm. The endoballoon was inflated above the proximal coronary anastomoses to occlude the aorta and control the bleeding. The endoballoon also served to deliver antegrade cardioplegia. In view of the patent LIMA‐LAD graft, continuous retrograde cardioplegia and systemic hyperkalemia also instituted and cardioplegic arrest was achieved; during this period the endoballoon served as a vent. Further dissection was carried out revealing a 1 cm defect in the distal ascending aorta at the previous cannulation site (Figure 4).

**Figure 5:**
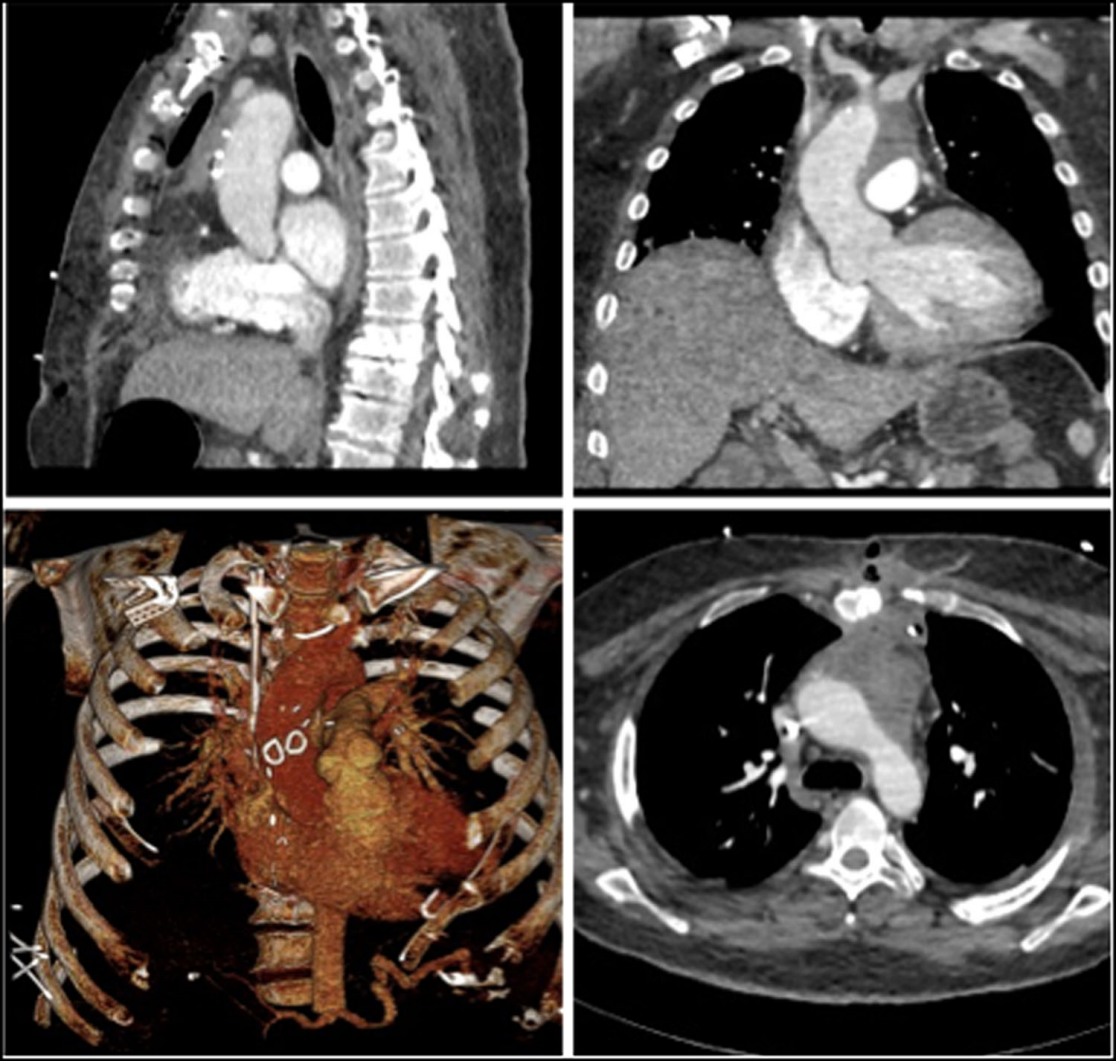
Computed Tomography images 2 days postoperatively demonstrating a normal aortic contour with resolution of the aortic pseudoaneurysm

Once the patient was adequately cooled, deep hypothermic circulatory arrest (DHCA) was instituted and the endoballoon deflated. A bovine pericardial patch was sewn into the defect, the aorta and heart de‐aired, systemic flow restored, and the patient rewarmed. Endoballoon inflation time was 32 min with DHCA of only 8 min. Postprocedural echo demonstrated normal biventricular function and the patient was transferred to intensive care in a stable condition. Post procedural CT demonstrated an excellent result with complete resolution of the pseudoaneurysm (Figure 5).

## 2 COMMENT

The development of minimally invasive mitral surgical approaches and the concomitant anesthetic upskilling that has been required has resulted in a broader range of tools at the disposal of cardiac surgical teams approaching cases such as these. Advantages of using the endoballoon in this setting are shorter DHCA time, excellent myocardial protection and complete decompression of the left ventricle during re‐entry. Whilst this approach has been reported in several other publications, this case is unique due to the added complication of patent coronary grafts^4^. Preprocedural CT imaging was utilized to identify an anatomical landmark which could be used on TEE to position the endoballoon relative to the patent grafts. The approach facilitated an excellent recovery. Knowledge of the balloon length is critical for precise placement and identification of a “landing zone” during cases with patent proximal coronary anastomoses. We believe the endoballoon strategy is easily replicated in centers with an active minimally invasive cardiac surgical program.
